# Survival outcomes and prognostic factors in surgically treated adult pilocytic astrocytomas

**DOI:** 10.3389/fonc.2025.1525427

**Published:** 2025-04-01

**Authors:** Sanghyeok Park, Jung Han Seo, Sang Woo Song, Young Hyun Cho, Chang-Ki Hong, Jeong Hoon Kim, Ho Sung Kim, Ji Eun Park, Soo Jung Nam, Young-Hoon Kim

**Affiliations:** ^1^ Department of Neurological Surgery, Asan Medical Center, University of Ulsan College of Medicine, Seoul, Republic of Korea; ^2^ Department of Radiology, Asan Medical Center, University of Ulsan College of Medicine, Seoul, Republic of Korea; ^3^ Department of Pathology, Asan Medical Center, University of Ulsan College of Medicine, Seoul, Republic of Korea

**Keywords:** pilocytic astrocytoma, adult, prognosis, gross total removal, neurological deficit, deep location

## Abstract

**Purpose:**

Pilocytic astrocytomas (PA) in adult patients are rare and the efficacy of postoperative adjuvant treatments remains unclear. This study aims to investigate the survival outcome and prognostic factors in surgically treated adult PA.

**Methods:**

A total of 90 consecutive adult patients with newly diagnosed PA were enrolled. Among the patients, 47 (52%) were male, with a median age of 28 years (18–70 years). Preoperative neurological deficits were observed in 43 (48%) patients. The most common tumor locations were cerebellar and cerebral hemispheres (28% and 27%, respectively), while 23% of tumors were located in deeper brain structures. The median follow-up duration was 88months (12–304 months).

**Results:**

Gross total removal (GTR) was achieved in 55 (61%) patients. At the final follow-up, 12 (13%) patients had died, and 23 (26%) experienced disease progression. The 1, 2, and 5-year overall survival (OS) rates were 93%, 91%, and 87%, respectively, while the progression-free survival (PFS) rates were 88%, 80%, and 77%, respectively. The recurrence rate in patients who underwent GTR was 11%, compared with 53% and 45% in those without GTR, with or without adjuvant treatments, respectively. The tumors in the deeper brain locations had significantly lower GTR rates (14%) compared with other locations (75%; *p* < 0.001). Multivariate analysis identified the absence of preoperative neurological deficits (*p* = 0.048; HR = 2.878), not deeper tumor location (*p* = 0.017; HR = 3.471) and GTR (*p* = 0.007; HR = 3.884) as significant factors for improved PFS.

**Conclusion:**

Adult PA exhibited more aggressive behavior compared with pediatric PA. These aggressive behaviors including preoperative neurological deficits, deeper tumor location, and lower GTR rates were significantly associated with poor prognosis.

## Introduction

Pilocytic astrocytomas (PA) are primary brain tumors classified as World Health Organization (WHO) grade 1 (1–3). They are the most common pediatric brain tumors in individuals aged 0–14 years and the second most common in those aged 15–19 years, accounting for 15.2% of all primary brain and central nervous system (CNS) tumors in individuals under 19 years. The incidence of PA in this age group is 0.95 per 100,000 person-years (4). In the pediatric population, PA typically occur in the cerebellum, whereas in adults, they are more frequently located in the cerebral hemisphere, followed by the cerebellum (5).

Despite their generally benign characteristics, some studies suggest that PA in adult patients may exhibit more aggressive behavior than in pediatric patients (5–8). However, Brown et al. suggested that PA in adults are as benign as those in children (9). The behavior of PA in adults remains poorly understood due to the rarity of the condition in this population, with an incidence of less than 0.1 per 100,000 person-years in individuals over 45 years old (4). The standard treatment for PA is surgical resection, with gross total removal (GTR) offering the most favorable prognosis. However, achieving GTR can be challenging, particularly for tumors located in deep regions such as the thalamus or brainstem. In such cases where GTR is not feasible, adjuvant radiation therapy (RTx) or stereotactic radiosurgery may be considered in some centers (10, 11). The efficacy of RTx in these situations, however, remains controversial (12, 13).

The aim of this study was to evaluate the survival outcomes of surgically treated adult PA and to identify prognostic factors in this patient population.

## Methods

### Study cohort

A total of 97 consecutive adult patients were diagnosed with primary PA between 1999 and 2022 at our institution. Inclusion criteria required patients to be aged 18 years or older, have histopathologically confirmed PA, a newly diagnosed primary PA, and a minimum clinical and radiological follow-up of 12 months. Two (2%) patients were excluded due to insufficient radiological data, and five (5%) were excluded due to loss of follow-up. Ultimately, 90 (93%) patients were enrolled in the study. All clinical information and neuroimaging data were collected with the approval of our institutional review board. This study was conducted in accordance with the Declaration of Helsinki, and informed consent was obtained from all participants.

### Radiological evaluations and treatment protocols

All patients underwent preoperative and postoperative magnetic resonance imaging (MRI) within 48 hours of surgery. Patients were followed for more than 6 months after surgery, with additional MRI evaluations to assess the extent of resection. The extent of resection was determined by comparing preoperative and postoperative MRIs. GTR was defined as no visible residual tumor on the immediate postoperative MRI. Subtotal removal (STR) was defined as less than 10% of the tumor remaining, while partial removal (PR) was defined as more than 10% of the tumor remaining on postoperative MRI. Biopsy was defined as obtaining tissue for histopathological examination (14).

At our institution, GTR was the primary surgical objective for suspected adult PA. If GTR was achieved, patients were monitored with follow-up imaging without additional treatment. For residual or recurrent tumors, reoperation was considered if feasible. If reoperation was not possible, follow-up observation was prioritized.

For deep-seated tumors with a high risk of progression or challenging reoperations, adjuvant therapy such as RTx, chemotherapy (CTx), or stereotactic radiosurgery (SRS) were considered. In cases of recurrence, salvage treatments, including reoperation, RTx, CTx or SRS, were performed.

### Outcome evaluation

Data were collected retrospectively by reviewing medical records and radiological findings. Collected variables included sex, age at diagnosis, presenting symptoms, presence of neurological deficits, tumor location, tumor size, infiltration, cyst components, gadolinium enhancement on brain MRI, perilesional edema, hydrocephalus, pathologic reports, extent of tumor removal, residual tumor size, and use of adjuvant or salvage treatments. The associations between clinical and radiological factors and progression-free survival (PFS) were analyzed.

### Statistical analysis

OS and PFS were the primary endpoints. OS was defined as the time from initial diagnosis to death, while PFS was defined as the time from initial treatment to tumor recurrence or progression, as determined by radiological findings. Cumulative rates of OS and PFS were evaluated using Kaplan–Meier survival methods. Prognostic factors for PFS were analyzed using logistic regression and Cox proportional hazard models, employing a backward stepwise method. To reduce the risk of type II errors due to the modest sample size, variables were included in the multivariate analysis only if they had a *p*-value of < 0.05 in univariate analyses. A *p*-value of < 0.05 was considered statistically significant. All statistical analyses were conducted using SPSS ver. 21.0 (SPSS Inc, Chicago, IL).

## Results

### Clinical and radiological characteristics

Among the 90 patients, 47 (52%) were male and 43 (48%) were female. The median age at diagnosis was 28 years old (range: 18–71 years), with 50 (56%) patients diagnosed before the age of 30. The most common preoperative symptoms were headache (54%), dizziness (31%), nausea and vomiting (23%), seizure (20%), visual disturbances such as diplopia (19%), gait disturbance (14%), and motor weakness (10%). Notably, three patients (3%) were asymptomatic. Forty-three (48%) patients presented with preoperative neurological deficits.

The most common tumor location was the cerebellar hemisphere (25 patients, 28%) followed by the cerebral hemisphere (24 patients, 27%), cerebellar vermis (15 patients, 17%), and lateral ventricle (5 patients, 5%). Twenty-one patients (23%) had tumors in deeper locations, such as the brainstem, third or fourth ventricle, basal ganglia, thalamus, pineal gland, or suprasellar area. The median tumor size was 4.0 cm (range: 1.2–8.0 cm), with 11 tumors (12%) showing infiltrative features. Thirty-seven patients (41%) presented with preoperative hydrocephalus, and 11 (12%) underwent external ventricular drainage or endoscopic third ventriculostomy prior to surgery. The median follow-up period was 88 months (range, 12–304 months). A summary of preoperative clinical and radiological characteristics is provided in **Table 1**.

**Table 1:** Baseline clinical and radiological characteristics of the study cohorts (n = 90).

Parameters	Values
Sex	
Male	47 (52%)
Female	43 (48%)
Median age at diagnosis (years)	28 (18 – 71)
Younger (< 30 years)	50 (56%)
Older (≥ 30 years)	40 (44%)
Initial symptoms	
Headache	49 (54%)
Dizziness	28 (31%)
Nausea and vomiting	21 (23%)
Seizure	18 (20%)
Visual disturbance (including diplopia)	17 (19%)
Gait disturbance	13 (14%)
Motor weakness	9 (10%)
No symptoms	3 (3%)
Preoperative neurological deficits	43 (48%)
Location of tumor	
Cerebellar hemisphere	25 (28%)
Cerebral hemisphere	24 (27%)
Cerebellar vermis	15 (17%)
Lateral ventricle	5 (6%)
Deeper location (brainstem, 3rd or 4th ventricle, basal ganglia, thalamus, pineal gland, or suprasellar area)	21 (23%)
Median size of tumors (cm)	4.0 (1.2 – 8.0)
Larger (≥ 4cm)	50 (56%)
Smaller (< 4cm)	40 (44%)
Infiltrative feature	11 (12%)
Mainly cystic tumor	33 (37%)
Strong enhancement on T1-weighted image	39 (43%)
Peritumoral edema	36 (40%)
Preoperative hydrocephalus	37 (41%)
Median follow-up duration (months)	88 (12 – 304)

### Surgical outcome

A summary of surgical and survival outcomes is presented in **Table 2**. Of the 90 patients who underwent surgery for PA, GTR was achieved in 55 (61%) patients. STR, PR, and biopsy were performed in 10 (11%), 12 (13%), and 13 (14%) patients, respectively. The median residual tumor was 2.8 cm (range: 1.0–5.6 cm). GTR rates highest in tumors located in the cerebral (88%) and cerebellar hemisphere (76%), whereas tumors in the deeper brain locations had significantly lower GTR rates (14%) compared with other locations (75%; *p* < 0.001). Infiltrative tumors (18%) and predominantly solid (51%) tumors had significantly lower GTR rates compared with circumscribed (67%) and cystic (79%) tumors (*p* = 0.003 and 0.013, respectively). Tumor size, enhancement, peri-tumoral edema, and hydrocephalus were not significantly associated with GTR rates (*p* = 0.384, 1.000, 0.270, and 0.278, respectively).

**Table 2:** Surgical and survival outcomes of adult patients with pilocytic astrocytomas (n = 90).

Parameters	Values
Extent of removal	
Gross total removal	55 (61%)
Subtotal removal	10 (11%)
Partial removal	12 (13%)
Biopsy	13 (14%)
Gross total removal according to tumor location	
Cerebellar hemisphere	19/25 (76%)
Cerebral hemisphere	21/24 (88%)
Cerebellar vermis	9/15 (60%)
Lateral ventricle	3/5 (60%)
Deeper location	3/21 (14%)
Median size of residual tumors (cm)	2.8 (1.0 – 5.6)
Adjuvant treatments for residual tumors	20/35 (57%)
Radiation therapy	12 (34%)
Reoperation	4 (11%)
Stereotactic radiosurgery	4 (11%)
Chemotherapy	2 (6%)
Progression after 1^st^ operation	23/90 (26%)
Salvage treatments for recurrent tumors	19/23 (83%)
Reoperation	11 (48%)
Chemotherapy	6 (26%)
Radiation therapy	5 (22%)
Stereotactic radiosurgery	5 (22%)
Progression after salvage treatments	12/23 (52%)
Death at final follow-up	12/90 (13%)
Progression free survival	
Mean (months)	223.0 (194.2 – 251.7)^*^
1-year rate (%)	87.8 (80.8 – 94.8) ^*^
2-year rate (%)	79.6 (71.0 – 88.2) ^*^
5-year rate (%)	77.1 (68.1 – 86.1) ^*^
Overall survival	
Mean (months)	257.5 (232.7 – 282.2) ^*^
1-year rate (%)	93.2 (87.8 – 98.6) ^*^
2-year rate (%)	90.8 (84.6 – 97.0) ^*^
5-year rate (%)	86.6 (78.2 – 95.0) ^*^

* (95% confidence interval).

### Adjuvant and salvage treatments

**Figure 1:**
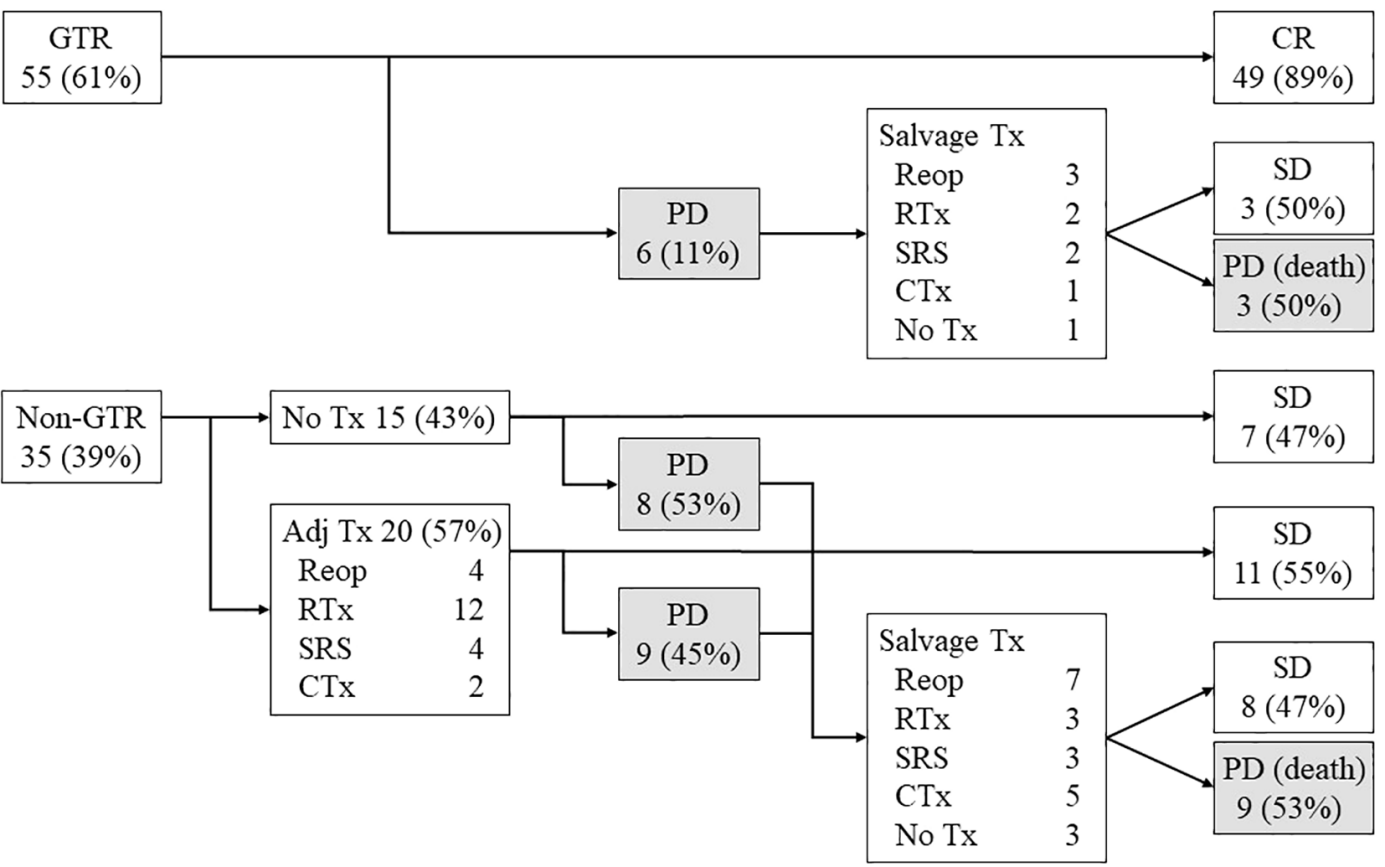
Treatment protocols and surgical outcomes of adult patients with pilocytic astrocytomas (n = 90). Among the 55 patients who underwent GTR, none received adjuvant treatments. Of the 35 patients who did not achieve GTR, 20 patients (57%) underwent adjuvant treatments. Tumor recurrence occurred in 6 patients (11%) after GTR, whereas 17 patients (49%) without GTR showed disease progression, regardless of adjuvant treatments (45% or 53%, respectively). GTR, gross total removal; Tx, treatments; Reop, reoperation; RTx, radiation therapy; SRS, stereotactic radiosurgery; CTx, chemotherapy; CR, complete remission; SD, stable disease; PD, progressive disease.

The outcomes associated with adjuvant or salvage treatments are depicted in **Figure 1**; **Table 2**. None of the 55 patients who underwent GTR received adjuvant treatments. Of the 35 patients who did not receive GTR, 20 (57%) received adjuvant therapy. Tumor recurrence occurred in 6 (11%) patients who had undergone GTR, compared with 17 (49%) of the 35 patients without GTR, regardless of whether they received adjuvant therapy (45% or 53%, respectively).

Among the 35 patients without GTR, four underwent reoperation, with only 1 (25%) showing disease progression. Of the remaining 31 patients without reoperation, 16 (52%) had tumor recurrence. After initial disease progression, 12 patients (52%) died due to disease progression, irrespective of the type of salvage. The treatment failure rates for recurrent tumors were 50% (5/10) for salvage surgery, 80% (4/5) for RTx, 40% (2/5) for SRS, and 67% (4/6) for CTx.

### Survival outcomes

During the follow-up period, 23 patients (26%) showed disease progression, and 12 (13%) died due to disease progression. The mean PFS was 223.0 months (95% confidence interval [CI], 194.2–251.7 months), with cumulative 1-, 2-, and 5-year PFS rates of 87.8%, 79.6%, and 77.1%, respectively. Mean OS was 257.5 months (95% CI, 232.7–282.2 months), with cumulative 1-, 2-, and 5-year OS rates of 93.2%, 90.8%, and 86.6%, respectively. The survival outcomes are summarized in **Table 2**.

### Prognostic factors

**Figure 2:**
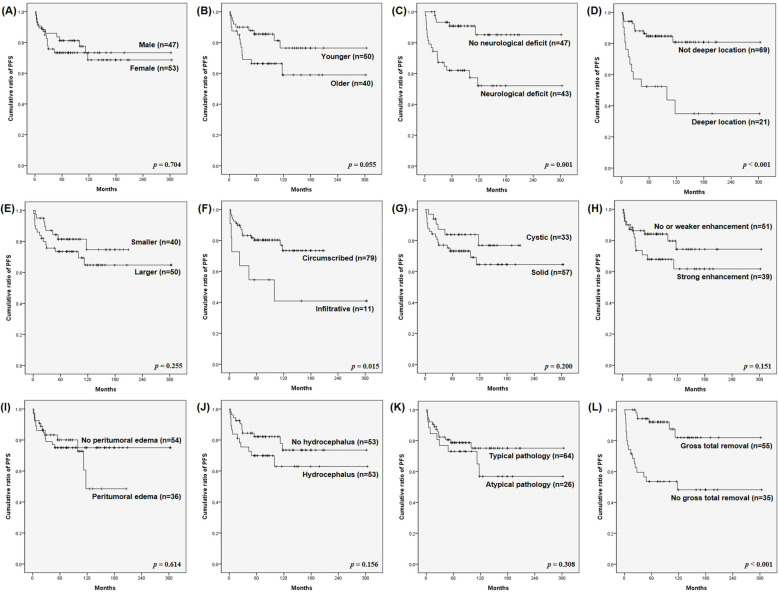
Kaplan–Meier survival curves for progression-free survival (PFS) of surgically treated adult pilocytic astrocytomas (PA). PFS were analyzed based on: **(A)** patient sex, **(B)** patient age, **(C)** preoperative neurological deficit, **(D)** tumor location, **(E)** tumor size, **(F)** infiltrative vs. circumscribed tumor pattern, **(G)** cystic vs. solid tumor, **(H)** enhancement pattern, **(I)** peritumoral edema, **(J)** hydrocephalus, **(K)** typical pathology, and **(L)** extent of resection. The **(C)** absence of preoperative neurological deficit, **(D)** non-deep tumor location, **(F)** circumscribed tumor pattern, and **(L)** gross total removal (GTR) were significant favorable prognostic factors for PFS of adult PA (*p* = 0.001, < 0.001, 0.015, and < 0.001, respectively).

Prognostic factors for PFS in adult patients with PA are analyzed in **Table 3**, and Kaplan–Meier PFS curves for various clinical and radiological factors are presented in **Figure 2**. Patient sex (**Figure 2A**), tumor size (**Figure 2E**), cystic morphology (**Figure 2G**), enhancement (**Figure 2H**), peri-tumoral edema (**Figure 2I**), preoperative hydrocephalus (**Figure 2J**), and pathology (**Figure 2K**) were not significantly associated with PFS (*p* = 0.704, 0.255, 0.200, 0.151, 0.614, 0.156, and 0.308, respectively). Patient age (**Figure 2B**) was marginally associated with PFS, but did not reach statistical significance (*p* = 0.055).

**Table 3:** Favorable prognostic factors for progression-free survival in adult patients who underwent surgical treatments for pilocytic astrocytomas (n = 90).

Factors	Progression-free survival (n = 90)		
	Univariate	Multivariate	
	*p-*value	*p*-value	HR (95% CI)
Sex (male)	0.704		
Younger patient (< 30 years)	0.055		
No preop neurological deficit	**0.001**	**0.049**	**2.878 (1.003 – 8.256)**
Not deeper location	**< 0.001**	**0.017**	**3.471 (1.250 – 9.635)**
Smaller tumor (< 4 cm)	0.255		
Circumscribed tumor	**0.015**	0.246	1.036 (0.372 – 2.889)
Cystic tumor	0.200		
No or weaker enhancement	0.151		
No peri-tumoral edema	0.614		
No hydrocephalus	0.156		
Typical pathology	0.308		
Gross total removal	**< 0.001**	**0.007**	**3.884 (1.440 – 10.475)**

HR, hazards ratio; CI, confidence interval; Preop, preoperative.

A p value < 0.05 is statistically significant.

In contrast, the absence of preoperative neurological deficits, non-deeper tumor location, circumscribed tumors, and GTR were significant favorable prognostic factors for PFS (*p* = 0.001, < 0.001, 0.015, and < 0.001, respectively). The 5-year PFS rates were significantly higher for patients without preoperative neurological deficits (**Figure 2C**), non-deep tumors (**Figure 2D**), circumscribed tumors (**Figure 2F**), and GTR (**Figure 2L**) (85.1%, 80.9%, 73.4%, and 82.0%, respectively) compared with those with preoperative neurological deficits, deep tumors, infiltrative tumors, and without GTR (52.1%, 34.9%, 40.9%, and 48.2%, respectively) (*p* = 0.001, < 0.001, 0.015, and < 0.001, respectively).

In the multivariate analysis, the absence of preoperative neurological deficits (p = 0.049; hazard ratio [HR] = 2.878; 95% CI, 1.003–8.256), not deeper tumor location (p = 0.017; HR = 3.471; 95% CI, 1.250–9.635) and GTR (p = 0.007; HR = 3.884; 95% CI, 1.440–10.475) were the only significant favorable prognostic factors.

## Discussion

### Surgical outcome

PA are WHO grade I primary brain tumors with a characteristic biphasic architecture, comprising densely packed fibrillary tissue and looser microcystic compartments. They typically display Rosenthal fibers and eosinophilic granular bodies (1, 3). PA are the most common primary brain tumor in the pediatric population (0–18 years), representing 15.2% of all primary CNS tumors in this age group (4). In the pediatric population, PA generally have an indolent nature and favorable prognosis, with 5-year OS rates nearing 100% and 10-year survival rates of 95.8% (12). However, adult PA are less well-characterized and can exhibit more aggressive behavior, with an incidence of less than 0.1 per 100,000 person-years in a population over 45 years (4). Stüer et al. reported a 30% tumor recurrence rate in adult PA, with 18% of patients succumbing to the disease over a 10-year follow-up period (15). Similarly, Theeler et al. reported a recurrence rate of 42% and reported 13 tumor-related deaths over a 22-year study of 127 adult patients with PA (6).

Our study aligns with these findings, with more than half of patients (50 of 90, 56%) diagnosed before the age of 30 years. Tumor locations were similar to those reported in other studies, with cerebellar hemisphere (28%), cerebral hemisphere (27%), and cerebellar vermis (17%) being the most common. According to the National Cancer Institute Surveillance data from 1973 to 2008, 27% and 30% of adult PA were located in the cerebellum and cerebrum, respectively (5). Our study reported a tumor progression rate of 26% (23 of 90) and a mortality rate of 13% (12 of 90), both significantly higher than in pediatric populations (5, 7, 16).

### Prognostic factors

In our study, younger patients exhibited relatively favorable PFS compared with older patients, although this difference was not statistically significant (*p* = 0.055). Previous studies have consistently shown that pediatric PA has a more favorable prognosis than adult PA (5, 6), with increasing age of diagnosis associated with more aggressive tumor behavior. Johnson et al. concluded that the varying prognosis across age groups is attributed to differences in PA aggressiveness (5). While age at diagnosis was a significant prognostic factor in their multivariate analysis, this was not the case in our study (5). This discrepancy may be attributed to differences in how patient age groups were categorized. Johnson et al. divided patients into several age brackets (under 5 years, under 20 years, under 40 years, under 60 years, and over 60 years) (5), which allowed them to demonstrate a prognostic impact of age. In contrast, we divided our cohort into just two groups: those under 30 and those over 30.

Tumor location and characteristics significantly impacted progression in our study, though these effects were largely due to the strong correlation between tumor location, features, and extent of resection. The three factors identified as significant factors for prognosis in this study, such as the presence or absence of preoperative neurological deficits, the location of the tumor, and the GTR, seem to be all related. In the case of tumors located deeper in the brain, the possibility of preoperative neurological deficits is high and the probability of GTR is significantly reduced. It can be seen that this aggressive behaviors of adult PA affects a poor prognosis.

GTR remains the most important factor influencing survival and prognosis in adult PA. Stüer et al. found that the recurrence rate for partially resected tumors was four times higher than for completely resected tumors (15). Similarly, Johnson et al. reported a significantly lower hazard ratio (0.3) for death in patients who underwent GTR compared with those who had STR or biopsy (5). Our results further confirm the positive impact of GTR on PFS. Even if GTR is not achieved during the initial surgery, reoperation with complete tumor resection can still yield favorable outcomes.

### Adjuvant and salvage treatments

In cases where GTR is not feasible, reoperation to achieve complete resection offers the best prognosis. However, when reoperation is not possible, adjuvant therapy, such as RTx, should be considered for patients with residual or recurrent tumors. In our study, the effects of RTx on residual or recurrent tumors did not show significantly better outcomes compared with other treatments or no treatment. The role of postoperative adjuvant RTx remains controversial, with conflicting reports regarding its efficacy. Ishkanian et al. reported superior PFS in patients who received adjuvant RTx in a retrospective analysis, supporting its use in adults (17). In contrast, Theeler et al. found significantly reduced PFS with adjuvant RTx (6).

The observed association between adjuvant RTx and higher progression rates is likely a result of patient selection bias, as RTx was primarily recommended for high-risk patients based on clinical, radiological, or pathologic factors. Further randomized prospective studies with larger populations are needed to elucidate the efficacy of adjuvant RTx for adult PA.

### Limitations of this study

The primary limitation of this study is its retrospective nature, conducted at a single institution. To account for the rarity of adult PA, we collected data over a 20-year period. During this time, surgical techniques and equipment, as well as diagnostic criteria, evolved. Consequently, there is a potential for misdiagnosis, particularly in earlier cases. In the current era, molecular diagnosis of brain tumors has gained prominence, and future studies will require detailed pathological analysis, including immunohistochemistry, to better establish prognostic factors for adult PA. For example, in the case of *KIAA1549-BRAF* fusion, which is expressed in 70-80% of pilocytic astocytoma, this mutation increases the possibility of gross total removal and makes it possible to predict a better prognosis. Therefore, molecular biological diagnosis of tumor tissue can be said to be an essential process for future glioma research (18).

## Conclusion

Adult PA tend to exhibit more aggressive behavior compared with pediatric PA. In our study, 26% of patients experienced tumor recurrence, and more than half of these patients died due to disease progression. The absence of preoperative neurological deficits, not deeper tumor location and GTR were the only significant prognostic factors associated with favorable PFS in adult PA. Further research is needed to better understand the efficacy and optimal selection of adjuvant or salvage treatments for adult PA.

## Data Availability

The original contributions presented in the study are included in the article/supplementary material. Further inquiries can be directed to the corresponding author.

## References

[1] Louis DN Perry A Reifenberger G Deimling Av Figarella−Branger D Cavenee WK. The 2016 world health organization classification of tumors of the central nervous system: a summary. Acta Neuropathol. (2016) 131:803–20. doi: 10.1007/s00401-016-1545-1 27157931

[2] Chen Y-H Gutmann D. The molecular and cell biology of pediatric low-grade gliomas. Oncogene. (2014) 33:2019–26. doi: 10.1038/onc.2013.148 23624918

[3] Sadighi Z Slopis J. Pilocytic astrocytoma: A disease with evolving molecular heterogeneity. J Child Neurol. (2013) 28:625–32. doi: 10.1177/0883073813476141 23439714

[4] Ostrom QT Cioffi G Waite K Kruchko C Barnholtz-Sloan JS. CBTRUS statistical report: primary brain and other central nervous system tumors diagnosed in the United States in 2014–2018. Neuro-Oncology. (2021) 23:iii1–iii105. doi: 10.1093/neuonc/noab200 34608945 PMC8491279

[5] Johnson DR Brown PD Galanis E Hammack JE. Pilocytic astrocytoma survival in adults: analysis of the Surveillance, Epidemiology, and End Results Program of the National Cancer Institute. J Neuro-oncol. (2012) 108:187–93. doi: 10.1007/s11060-012-0829-0 22367412

[6] Theeler BJ Ellezam B Sadighi ZS Mehta V Tran MD Adesina AM. Adult pilocytic astrocytomas: clinical features and molecular analysis. Neuro-oncology. (2014) 16:841–7. doi: 10.1093/neuonc/not246 PMC4022218 24470550

[7] Yang W Porras JL Khalafallah AM Sun Y Bettegowda A Mukherjee D. Comparison of adult and pediatric pilocytic astrocytomas using competing risk analysis: A population-based study. Clin Neurol Neurosurg. (2022) 212:107084. doi: 10.1016/j.clineuro.2021.107084 34875553

[8] Muhsen BA Aljariri AI Elayyan M Hirbawi H Masri MA. Insight about the characteristics and surgical resectability of adult pilocytic astrocytoma: tertiary center experience. CNS Oncol. (2022) 11:CNS81. doi: 10.2217/cns-2021-0014 PMC8988253 35382555

[9] Brown PD Anderson SK Carrero XW O’Neill BP Giannini C Galanis E. Adult patients with supratentorial pilocytic astrocytoma: long-term follow-up of prospective multicenter clinical trial NCCTG-867251 (Alliance). Neuro-oncol Pract. (2015) 2:199–204. doi: 10.1093/nop/npv031 PMC4669035 26640699

[10] Trifiletti DM Peach MS Xu Z Kersh R Showalter TN Sheehan JP. Evaluation of outcomes after stereotactic radiosurgery for pilocytic astrocytoma. J Neurooncol. (2017) 134:297–302. doi: 10.1007/s11060-017-2521-x 28567590

[11] Hafez RFA Fahmy OM Hassan HT Ganz JC. Gamma Knife Radiosurgery for symptomatic eloquently deep-seated cystic pilocytic astrocytoma mural nodules: Retrospective case series of effective outcomes. Acta Neurochir (Wien). (2024) 166:466. doi: 10.1007/s00701-024-06366-7 39565484

[12] Burkhard C Patre P-LD Schüler D Schüler G Yas¸Argil MG Yonekawa Y. A population-based study of the incidence and survival rates in patients with pilocytic astrocytoma. J Neurosurg. (2003) 98:1170–4. doi: 10.3171/jns.2003.98.6.1170 12816259

[13] Khalafallah AM Jimenez AE Shah PP Brem H Mukherjee D. Effect of radiation therapy on overall survival following subtotal resection of adult pilocytic astrocytoma. J Clin Neurosci. (2020) 81:340–5. doi: 10.1016/j.jocn.2020.10.020 33222942

[14] Byun J Kim YH Nam SJ Park JE Cho YH Kim HS. Comparison of survival outcomes between partial resection and biopsy for primary glioblastoma: A propensity score-matched study. World Neurosurg. (2019) 121:e858–66. doi: 10.1016/j.wneu.2018.09.237 30315970

[15] Stüer C Vilz B Majores M Becker A Schramm J Simon M. Frequent recurrence and progression in pilocytic astrocytoma in adults. Cancer. (2007) 110:2799–808. doi: 10.1002/cncr.v110:12 17973253

[16] Kim JW Phi JH Kim SK Lee JH Park SH Won JK. Comparison of the clinical features and treatment outcomes of pilocytic astrocytoma in pediatric and adult patients. Childs Nerv Syst. (2023) 39:583–91. doi: 10.1007/s00381-023-05839-x 36662276

[17] Ishkanian A Laperriere NJ Xu W Millar B-A Payne D Mason W. Upfront observation versus radiation forAdult pilocytic astrocytoma. Cancer. (2011) 117:4070–9. doi: 10.1002/cncr.v117.17 21391213

[18] Ryall S Tabori U Hawkins C. Pediatric low-grade glioma in the era of molecular diagnostics. Acta Neuropathol Commun. (2020) 8:30. doi: 10.1186/s40478-020-00902-z 32164789 PMC7066826

